# Advances in nutritional supplementation for sarcopenia management

**DOI:** 10.3389/fnut.2023.1189522

**Published:** 2023-07-10

**Authors:** Simin Liu, Lin Zhang, Shuangqing Li

**Affiliations:** ^1^General Practice Ward/International Medical Center Ward, General Practice Medical Center, West China Hospital, Sichuan University, Chengdu, Sichuan, China; ^2^National Clinical Research Center for Geriatrics, Multimorbidity Laboratory, West China Hospital, Sichuan University, Chengdu, Sichuan, China

**Keywords:** sarcopenia, nutritional supplements, protein, leucine, β-hydroxy-β-methylbutyric acid, antioxidant, omega-3 fatty acids, creatine

## Abstract

Sarcopenia is a syndrome characterized by a decline in muscular mass, strength, and function with advancing age. The risk of falls, fragility, hospitalization, and death is considerably increased in the senior population due to sarcopenia. Although there is no conclusive evidence for drug treatment, resistance training has been unanimously recognized as a first-line treatment for managing sarcopenia, and numerous studies have also pointed to the combination of nutritional supplementation and resistance training as a more effective intervention to improve quality of life for people with sarcopenia. People with both malnutrition and sarcopenia have a higher mortality rate, so identifying people at risk of malnutrition and intervening early is extremely important to avoid sarcopenia and its associated problems. This article provides important information for dietary interventions in sarcopenia by summarizing the discoveries and developments of nutritional supplements such as protein, leucine, β-hydroxy-β-methylbutyric acid, vitamin D, vitamin C, vitamin E, omega-3 fatty acids, creatine, inorganic nitrate, probiotics, minerals, collagen peptides, and polyphenols in the management of sarcopenia.

## 1. Introduction

After the age of 50, muscle mass declines at a rate of 1%−2% per year in healthy adults, while muscle strength declines at a rate of 1.5% per year (1, 2). Sarcopenia was defined by the European Working Group on Sarcopenia in Older People (EWGSOP) in 2010 as “a syndrome characterized by progressive and generalized loss of skeletal muscle mass and strength with a risk of adverse outcomes such as physical disability, poor quality of life, and death” (3). In 2018, the EWGSOP updated the definition, and muscle strength and function are now put in front because the two are more important than muscle mass (4). Sarcopenia is a major public health concern because it affects 20% of people over the age of 70 and 50% of people over the age of 80 (5). Currently, 6%−19% of the global population over the age of 60 suffers from sarcopenia (6).

Sarcopenia should be suspected in patients who present with signs or symptoms such as falls, feeling weak, walking slowly, difficulty rising from a chair, weight loss, or muscle wasting, and EWGSOP2 recommends screening and evaluation starting with the Simple Five Item Scoring Questionnaire (SARC-F) (4). When low muscle strength is tested by grip strength or chair stand, it is considered very likely to have sarcopenia, because according to EWGSOP, the diagnosis of sarcopenia is “low muscle mass and low muscle function (strength or performance),” while muscle strength is the center of the diagnostic process (3). The Asian Working Group for Sarcopenia (AWGS) 2019 consensus defined “probable sarcopenia” as low muscle strength or physical fitness, specifically used in primary care or community-based health promotion (7). In addition, low muscle mass can be measured by instruments such as DXA, BIA, CT, and MRI. The severity of sarcopenia can be determined by measuring physical performance, such as gait speed, SPPB, TUG, and 400 m walk (4). Ideally, a primary care physician should begin screening older adults at risk for sarcopenia, then diagnose them through the use of appropriate diagnostic tools, and begin treatment as early as possible. This will prevent delays in the diagnosis and management of sarcopenia. Common tools for screening and diagnosing sarcopenia and their respective advantages and disadvantages are shown in Table 1.

**Table 1:** Tools for screening and diagnosing (7, 8).

	**Category**	**Disadvantages**	**Advantages**
Screening tools	SARC-F questionnaire	Low to moderate sensitivity	Quick and easy to use
	Anthropometry	Measurement variability, lack of international standardization	Cheap, easy to perform
Diagnostic tools	Computed tomography (CT)	Gold standard for skeletal muscle mass	Expensive, high radiation
	Magnetic resonance imaging (MRI)	Cross-sectional analysis of muscle quantity and mass, no radiation	High cost, complex operation, time-consuming
	DXA (dual-energy X-ray absorptiometry)	Precise analysis of body composition, very low radiation exposure, operational	High cost
	Bioelectrical Impedance Analysis (BIA)	Accurate, inexpensive, simple, safe	Susceptible to fluid changes, etc.
	Ultrasound	inexpensive, simple, safe	Measurement accuracy to be verified

Anthropometry: Body mass index (BMI), mid-upper arm circumference (MUAC), calf circumference (CC), triceps skinfold (TSF).

It has been reported that older residents at risk of malnutrition in Asian communities range from 16 to 73% (9), and the combined prevalence of moderate to high malnutrition risk among elderly people in Europe is 48.4% (10). In Latin America, two out of every five hospitalized patients are at risk of malnutrition (11). Malnutrition increases the risk of sarcopenia by two to three times, and people with sarcopenia who are undernourished have a higher mortality rate (12, 13). According to the AWGS consensus, older adults who present with low body mass index (BMI), unintentional weight loss, and low muscle mass or exhibit poor muscle strength at any time should be assessed for malnutrition, and those at risk of malnutrition should be rescreened every 3 months (9). Some evidence has shown that adequate intake of protein, vitamin D, antioxidant nutrients, and long-chain polyunsaturated fatty acids is beneficial for improving sarcopenia (14). Healthcare professionals are advised to provide nutritional counseling on dietary habits for older adults with sarcopenia and to collaborate with a dietitian to develop a diet and protein optimization plan for the patient (15). Therefore, identifying individuals at risk for malnutrition to provide early intervention is an important public health strategy to prevent the development of sarcopenia and related complications.

## 2. Nutritional intervention for sarcopenia

### 2.1. Protein, leucine, β-hydroxy-β-methylbutyric acid

#### 2.1.1. Mechanisms

On the metabolic front, temporal fluctuations in muscle protein synthesis (MPS) and muscle protein breakdown (MPB) rates determine the net increase or decrease in skeletal muscle protein, which continuously remodels skeletal muscle mass. Protein degradation exceeding protein synthesis results in a negative nitrogen balance and triggers sarcopenia. Dietary protein or amino acid intake is the primary physiological stimulus for MPS (16), stimulating muscle protein synthesis, increasing muscle mass, and reducing muscle loss during bed rest and aging (17).

First, the ubiquitin–proteasome system (UPS) is the major pathway for cellular protein degradation, and studies have demonstrated that activating the UPS leads to increased protein degradation and finally results in sarcopenia (18, 19), while protein or amino acid nutritional support can effectively downregulate the levels of MuRF-1 and Atrogin-1 and ameliorate UPP-mediated sarcopenia (20, 21). Second, the oxidative response is one of the inducers of sarcopenia (22). Protein or amino acid nutritional support contributes to promoting Sirt1 expression, activating FoxO family proteins, enhancing the expression of SOD, and reducing the oxidative response (23). Third, enhanced autophagy evokes sarcopenia (24). Protein or amino acid nutritional support can enhance the activity of the PI3K/Akt/mTOR signaling pathway to suppress cell autophagy (25). There are some potential new mechanisms, including altering miRNA profiles and gut microbiota (26).

#### 2.1.2. Clinical studies

##### 2.1.2.1. Protein

To measure the effect of protein supplementation on muscle health, Hanach et al. analyzed 14 RCTs (27), with milk protein or protein-based dairy products for not <12 weeks as the intervention. The results showed that milk protein significantly increased limb muscle mass, although there was no effect on grip strength or leg muscle strength, and there was no conclusive evidence of an effect on physical activity. Kirwan et al. (28) analyzed 28 RCTs and showed that among older adults who performed resistance training, those who consumed higher protein increased lean limb mass and grip strength compared to controls who were supplemented with lower protein; however, without resistance training, there was no additional benefit from protein supplementation alone. In healthy older adults from Asia and other countries, the combination of protein supplementation and exercise significantly increased lower extremity strength compared to exercise alone or placebo, although no significant differences were found in upper extremity strength, muscle mass, or gait speed (29). Therefore, it is recommended to supplement protein in combination with resistance training to increase muscle mass and strength (30). Regarding the relationship between dietary protein intake and skeletal muscle mass, a cross-sectional analysis of 3,213 middle-aged and elderly residents in the mainland Chinese community found that participants who consumed more than 0.96 g/kg of protein per day had higher muscle mass than those who consumed no more than 0.96 g/kg of protein per day (31). In elderly subjects aged 70–85 years, those who consumed 1.5 g/(kg/day) of protein continuously for 12 weeks had higher skeletal muscle mass and mass index and higher gait speed, while the other two groups (0.8 and 1.2 g/kg/day of protein, respectively) did not differ significantly in terms of muscle mass and physical performance (32). A dose-dependent increase in whole-body net protein balance during recovery from resistance exercise in older healthy men randomly assigned to consume 0 g, 15 g, 30 g, or 45 g of milk protein concentrate suggests that the dose of protein consumed after exercise is a key factor in the magnitude of the muscle protein synthesis response (33). The World Health Organization and the U.S. The National Academy of Sciences currently recommends a protein daily allowance (RDA) of 0.8 g/kg/day for adults, but this value applies to all ages, regardless of gender, physical activity, or health status. Evidence from RCTs in elderly populations, as well as the protein requirements of elderly individuals measured using the indicator amino acid oxidation (IAAO) technique, suggests that this dose does not meet the physiological protein requirements of elderly individuals. The estimated average protein requirement (EAR) and RDA measured using IAAO technology were 0.94 and 1.24 g/kg/day in older men and 0.96 and 1.29 g/kg/day in older women, respectively (34, 35). According to the European Society of Clinical Nutrition and Metabolism (ESPEN), the diet of the elderly should provide at least 1.0–1.5 g protein/kg body weight/day, with 25–30 g protein allocated to each meal (36). However, patients with severe chronic kidney disease should limit their protein intake. In summary, most studies confirm that protein intake is positively correlated with muscle mass and strength and that higher protein intake has a positive effect on skeletal muscle health during aging. However, protein supplementation is not recommended as an independent intervention to improve muscle mass and strength. Protein supplementation only during resistance training can significantly improve grip strength and physical function, and the combination of the two can improve sarcopenia significantly more than resistance training alone (37–39). There is bias and heterogeneity in the evidence for protein supplementation on measures of muscle mass and the effects of strength and physical performance, and differences in the type and dose of protein supplementation, as well as variations in exercise regimen and duration, need to be taken into account when interpreting the results. More carefully designed large-scale randomized controlled trials exploring the effects of protein supplementation on these measures are needed in the future.

The quality and digestibility of proteins are distinguishing features between animal and plant proteins, with differences in amino acid content and absorption kinetics. Animal proteins such as meat, fish, and dairy products are consistently high-quality proteins, while plant proteins vary in quality depending on the sources, with soy protein being recognized as a high-quality plant protein. Therefore, with respect to the quality of protein, animal-derived protein may be more effective in maintaining muscle health. Regarding potential differences between animal and plant proteins affecting muscle health, a meta-analysis of 16 RCTs (51) showed that protein sources did not affect changes in absolute lean body mass or muscle strength; however, animal protein was more beneficial for percent lean body mass. It was shown in a retrospective study that men and women with higher animal protein intake had higher percentages of skeletal muscle mass regardless of physical activity, while the beneficial effects of plant protein were only shown in physically active adults (52). In contradiction to these findings, a negative correlation between walking speed and relative animal protein intake and a positive correlation with relative plant protein intake have also been reported (53). Gazzani et al. (54) also supported the positive effect of plant proteins on physical performance and suggested that this could be related to other components of plant foods that affect muscle mass and strength, such as antioxidants. It is unclear how protein intake from different sources provides the best benefit for preventing sarcopenia, and further research is needed to refine protein dietary guidelines that promote muscle health.

##### 2.1.2.2. Leucine

Amino acids are important raw materials for protein synthesis, and their homeostasis is essential for maintaining muscle health. Muscle protein synthesis is regulated at multiple physiological levels, including dietary protein digestion and amino acid absorption, visceral amino acid retention, postprandial insulin release, skeletal muscle tissue perfusion, muscle uptake of amino acids, and intracellular signaling in myocytes (55). Therefore, some scholars have proposed that the anabolic potential of proteins correlates with amino acid composition, which is supported by the finding that plasma concentrations of leucine, isoleucine, and tryptophan are reduced in patients with sarcopenia (56). Synthesis by activating rapamycin complex 1 (mTORC1), a target that acts as a “switch” for the MPS process, which initiates translation in the intracellular signaling cascade (57). Thus, the “leucine trigger” hypothesis has been proposed, which predicts that the magnitude and rate of postprandial blood leucine increase may modulate the magnitude of the postprandial MPS response to protein intake. Sixteen of the 29 eligible studies provided sufficient evidence to support the hypothesis (58). Thus, leucine content may be a key factor in promoting the muscle protein synthesis response. The effect of 25 g of whey protein on maintaining skeletal muscle protein synthesis and improving muscle loss is similar to that of 10 g of milk with leucine in older adults (59). Compared to isonitrogenous protein drinks, protein drinks with higher concentrations of leucine are more beneficial for myogenic fibronectin synthesis (60). Leucine supplementation has been reported to have beneficial effects on body weight, body mass index, and lean body mass in older adults with a tendency toward sarcopenia, although the effects on muscle strength are inconclusive (61). Besides, according to a systematic review, protein supplements rich in leucine can improve markers of sarcopenia, regardless of physical activity, however, leucine supplementation alone and no exercise did not improve sarcopenia (62). Current evidence tends to recommend a higher intake of leucine in older adults to increase muscle mass. Considering the importance of leucine in muscle protein synthesis, leucine requirements in elderly individuals, measured using the indicator amino acid oxidation method, are more than twice the current recommendations, averaging 77.8 mg/(kg/day) for men and 78.2 mg/(kg/day) for women (63).

##### 2.1.2.3. β-hydroxy-β-methylbutyric acid

β-hydroxy-β-methylbutyric acid (HMB) is a metabolite of leucine. The International Society of Sports Nutrition believes that HMB can reduce exercise-induced skeletal muscle damage and is most effective when consumed for two consecutive weeks before exercise, so athletes are recommended to take 38 mg per kg of body weight per day to promote their skeletal muscle growth and improve strength (64). The manufacturer usually recommends taking 3 g of HMB per day (40), the dose being equivalent to the intake of 60 g of leucine (65). However, if 60 g of leucine is consumed directly, the activity of rate-limiting enzymes for catabolism increases, and the oxidation of branched-chain amino acids increases, which can lead to depletion of valine and isoleucine in body fluids and ultimately an imbalance in the concentration of branched-chain amino acids, thus possibly having a negative impact on protein metabolism (66); however, there is wide heterogeneity in the conclusions drawn from published articles regarding the effects of HMB supplementation on muscle health and physical performance. Supporting research findings indicate that HMB intake promotes both upper and lower-extremity muscle strength in older adults (67). Supplementation with 3 g of HMB is most beneficial for improving strength and body composition in people over 65 years of age, especially when bed-rested and untrained (68). In the opposing study, Phillips et al. (69) stated through systematic evaluation and meta-analysis that the current evidence is insufficient to assess the effects of HMB supplementation on muscle function, as the evidence supports little and is inconsistent. In a randomized controlled trial carried out among 40 young adult men (70), the intervention group was supplemented with the leucine metabolites alpha-hydroxyisocaproic acid (α-HICA) and β-hydroxy-β-methylbutyric acid (HMB); as a result, supplementation with leucine metabolites did not enhance resistance training-induced changes in muscle thickness compared to placebo (71). In conclusion, more high-quality primary studies are needed in the future to investigate the effects of HMB in patients with sarcopenia, and the current evidence does not yet provide unambiguous support for recommending HMB supplementation to alleviate sarcopenia.

### 2.2. Vitamins

#### 2.2.1. Vitamin D

##### 2.2.1.1. Mechanisms

The vitamin D/VDR axis plays a key role in regulating biological processes central to sarcopenic muscle atrophy, such as proteolysis, mitochondrial function, cellular senescence, and adiposity (72). First, vitamin D deficiency appears to lead to increased muscle protein breakdown via the ubiquitin-proteasomal pathway (UPP) and autophagy and upregulation of AMPK and members of the renin-angiotensin system (73, 74). Second, permanent exit from the cell cycle (senescence) is a critical aging phenomenon, and the vitamin D/VDR axis has been shown to have regulatory control (75). Third, low vitamin D states may lead to impaired mitochondrial function (76), and active 1,25(OH)_2_D3 can increase oxygen consumption rates and fission/fusion dynamics (77, 78). Fourth, low vitamin D states may lead to increased adiposity in muscle (79), and those who are overweight have an increased risk of deficits in muscle mass and function (80).

##### 2.2.1.2. Clinical studies

Vitamin D is a fat-soluble vitamin synthesized in the skin, 90% of which comes from UV exposure and 10% from the diet. Vitamin D deficiency is now considered a global public health problem, and elderly individuals are at greater risk of vitamin D deficiency due to poor intestinal absorption, reduced sun exposure, and chronic renal insufficiency. Lower 25-(OH)-VD levels are thought to be associated with adverse changes in muscle mass and physical function (81). Yang et al. (82) fed mice a vitamin D-deficient diet for 24 weeks and immobilized them to determine the extent of skeletal muscle atrophy. As a result, vitamin D deficiency accelerated the decrease in gastrocnemius muscle mass, muscle fiber cross-sectional area, and grip strength; moreover, vitamin D supplementation inhibited the decrease in grip strength. The team also performed a cross-sectional analysis of 4,139 older adults, and linear regression analysis showed that serum 25 hydroxyvitamin D and physical activity were linearly associated and interacted with timed running time and grip strength. However, in another study in which the control group took a placebo daily and the intervention group took 800 IU of vitamin D orally daily, no differences were found between the two groups in leg push-up strength, function, or lean body mass after 1 year (83). According to the systematic reviews and meta-analyses, vitamin D supplementation alone did not improve muscle strength or SPPB scores, on the contrary, significantly decreased SPPB scores (84). When vitamin D was taken together with whey protein and leucine, the muscle mass of the limbs of patients with sarcopenia could be effectively increased even without physical exercise, and when combined with physical exercise, not only muscle mass increases but muscle strength and performance could also be significantly improved (85). However, we cannot be sure of the effectiveness of vitamin D supplementation alone, due to the presence of protein and amino acids. In summary, the exact role of vitamin D supplementation in the prevention and treatment of sarcopenia remains uncertain due to the high heterogeneity of studies and the conflicting results of RCTs.

#### 2.2.2. Vitamin C and vitamin E

##### 2.2.2.1. Mechanisms

With aging, the body's endogenous antioxidant defense system is impaired, and excessive accumulation of reactive oxygen species (ROS) in the body leads to oxidative muscle damage, which may be directly or indirectly involved in skeletal muscle atrophy (86). In addition, mitochondrial dysfunction occurs abnormally during muscle aging, which has been associated with aberrant ROS generation and oxidative damage (87). Antioxidant vitamins are thought to prevent oxidative stress and may be able to play a role in the treatment of sarcopenia. Therefore, whether antioxidant supplementation can improve age-related muscle mass and performance is becoming an issue of interest to researchers. Vitamins C and E are widely used antioxidant vitamins that have the ability to scavenge ROS and enhance cellular antioxidant capacity.

##### 2.2.2.2. Related studies

Vitamin E is composed of two subgroups called tocopherols and tocotrienols. There are four isomers of tocopherols and tocotrienols (α, β, γ, and δ) depending on the number and location of the methyl groups, and their main dietary sources are vegetable oils, nuts, seeds, fish, shellfish, and vegetables (88). *In vitro*, studies have shown that alpha-tocopherol prevents myogenic cell atrophy and increases myotube survival (89), and the tocotrienol-rich fraction reverses the aging of myogenic cells by increasing the regenerative capacity of cells (90). Vitamin E contributes to the recovery of myogenic cell membranes and has a potential therapeutic effect on muscle cells (91), although further studies are needed to confirm the mechanisms involved. In a cross-sectional study, a significant positive association was found between increased dietary vitamin E intake and skeletal muscle mass index, bone mineral density status, and risk of total hip and hip fracture in middle-aged and older men and women, with effects ranging from 0.88 to 1.91% (92).

Vitamin C is the major water-soluble nonenzymatic antioxidant in plasma and tissues and must be obtained through dietary intake because it cannot be synthesized *in vivo*. A positive trend in quintiles of dietary vitamin C and lean body mass measurements suggests that dietary and circulating vitamin C is positively associated with skeletal muscle mass measurements in middle-aged and older men and women (93).

However, contrary studies have also been reported. In a systematic evaluation and meta-analysis, vitamins C and E did not promote muscle growth after strength training and may have diminished muscle hypertrophy over time (94). When young athletes were given vitamin C and E supplements, despite serum samples suggesting a reduction in oxidative stress in the body, participants' lower limb strength did not increase, and muscle damage could not be reduced (95). In summary, based on the existing evidence, there is not enough convincing evidence to support the use of vitamin E and vitamin C for the prevention and treatment of sarcopenia.

### 2.3. Omega-3 fatty acids

Omega-3 fatty acids (also known as n-3 fatty acids) are polyunsaturated fatty acids with many potential health benefits and are available in three main dietary forms: alpha-linolenic acid (ALA; 18:3n-3), eicosapentaenoic acid (EPA; 20:5n-3) and docosahexaenoic acid (DHA; 22:6n-3). ALA is considered an essential fatty acid because it cannot be synthesized in the human body and is found in nuts, seeds, canola oil, etc. EPA and DHA are mainly found in fish oil.

#### 2.3.1. Mechanisms

Skeletal muscle atrophy involves an inflammatory phase that leads to cell death and tissue remodeling and activates endoplasmic reticulum stress (ERS) and autophagy (96, 97). Both EPA and DHA potentially attenuate ERS and autophagy in skeletal muscles undergoing atrophy by attenuating the increase in PERK and ATG14 expression (98). In addition, DHA promotes mitochondrial biogenesis and skeletal muscle fiber remodeling (99) and delays muscle wasting by stimulating intermediate oxidative stress and inhibiting proteasomal degradation of muscle proteins (100).

#### 2.3.2. Related studies

It has been proposed that elevated plasma levels of proinflammatory cytokines affect muscle catabolic and anabolic signaling pathways and thus may play a key role in the development and progression of sarcopenia, with data showing significantly elevated levels of IL-6 and TNFα in elderly Chinese individuals with sarcopenia (101). Therefore, reducing chronic inflammation associated with aging is emerging as a potential therapeutic target for sarcopenia, and NSAIDs may not be recommended for the treatment of sarcopenia due to the high risk of adverse events that may occur with their use in elderly individuals. Increasing evidence indicates that omega-3 polyunsaturated fatty acids reduce the expression of inflammatory genes and have anti-inflammatory activity (102), particularly eicosapentaenoic acid, docosahexaenoic acid, and alpha-linolenic acid. It was concluded from a systematic evaluation and meta-analysis that omega-3 fatty acid supplementation promotes lean body mass, skeletal muscle mass, and isometric contraction maximal muscle strength in the quadriceps (103). Dietary omega-3 fatty acid levels are negatively associated with sarcopenia (104), and more than 2 g/day of omega-3 fatty acids may increase muscle mass and improve walking speed, especially for those with sarcopenia who have been receiving the intervention for more than 6 months (105). However, linear regression analysis concluded that there was no association between plasma omega-3 levels and grip strength in older adults (106). When cancer patients were supplemented with omega-3 fatty acids, their muscle maintenance, quality of life, and body weight were not improved (107). According to expert opinions (108), doses of 3,000 mg/day DHA plus EPA or more (with preferably more than 800 mg/day EPA) may be required for positive physical performance in older adults (109, 110), because lower doses have no significant effects on muscle strength (111). In conclusion, omega-3 fatty acids may improve sarcopenia, but well-designed, large prospective cohort studies and randomized controlled trials are needed to confirm these findings.

### 2.4. Creatine

Creatine is a natural nonprotein amino acid compound. Approximately half of the daily creatine requirement comes from the diet, mainly in red meat and seafood (112), and the other half is synthesized endogenously in the kidneys and liver (113). Creatine is mainly stored in muscle (95%), ~2/3 is in the form of PCr, and the rest is free creatine. Approximately 1%−2% of intramuscular creatine is degraded to creatinine and excreted in the urine each day (114, 115); therefore, the body needs to replenish ~1–3 g of creatine per day to maintain normal creatine stores and to obtain the free energy provided by catabolism, depending on muscle mass (116).

#### 2.4.1. Mechanisms

The energy produced by phosphocreatine (PCr) degradation is used to resynthesize ADP and Pi back into ATP to maintain cell function. Increasing PCr and creatine in muscles provides energy reserves to meet anaerobic energy needs, providing a critical source of energy, especially during ischemia, injury, and/or response to impairment (117, 118). Creatine has been shown to activate signaling pathways in the muscle protein synthesis pathway (119), and creatine also protects against mitochondrial damage caused by oxidation, which may reduce inflammation and muscle damage (120, 121).

#### 2.4.2. Clinical studies

After examining the effects of different creatine dosing strategies (lower: 5 g/day, higher: >5 g/day) and the presence or absence of a creatine loading phase (20 g/day for 5–7 days) on lean tissue mass and strength, overall, creatine increased lean tissue mass and strength, but when studies involving a creatine loading phase were excluded from the analysis, creatine had no greater benefit on muscle mass and strength compared to placebo and was effective only during the resistance training phase (122). In another study, creatine supplementation significantly increased upper extremity strength but had no effect on lower extremity strength or muscle mass. However, when resistance training was continued for at least 24 weeks, a significant increase was found in upper and lower extremity muscle strength among older females (123). Overall, creatine intake during resistance training in older adults may increase lean tissue mass, as well as muscle strength in the upper and lower extremities (124). Therefore, it is recommended that older adults supplement creatine concurrently with resistance training. Creatine supplementation appears to enhance the muscular adaptive response to training by increasing the ability to exercise at high intensities and enhancing postexercise recovery and adaptation (125). Differences in creatine dose and frequency of intake during resistance training need to be considered when interpreting the heterogeneity between these studies (126).

### 2.5. Inorganic nitrate

The health benefits of a diet rich in vegetables are partly explained by their high nitrate content, which is an important biologically active cardioprotective component of vegetables due to its effects on endogenous nitric oxide and vascular health (127). Approximately 80% of total dietary nitrate intake comes from vegetables, with leafy greens and beet being the most abundant and the rest from fruits and meat (128).

Skeletal muscle tissue is the largest reservoir of nitrate in the body and one of the main sites of nitrate and nitrite metabolism (129), which is sensitive to dietary nitrate intake, contributing to nitric oxide production during exercise, and it enhances human muscle contraction by increasing the free intracellular calcium concentration and the calcium sensitivity of myofilaments themselves (130, 131). A cross-sectional analysis revealed that higher nitrate intake (mean 31.2 mg/day) is associated with stronger grip strength and faster timed runs (132). Researchers evaluated participants' habitual dietary intake over 12 years in a cohort study, and individuals with the highest nitrate intake (mean 91 mg/day) had stronger knee extension and faster timed starts, and the results were unaffected by physical activity (133). In randomized controlled trials, nitrate is given almost in the form of concentrated beetroot as an acute dose ranging from 6.4 to 15.9 mmol, and the results show that $NO_{3}^{-}$ intake significantly increases muscle strength, with an average increase of ~5% (134). Its potential benefits on muscle strength and endurance are not affected by dose, frequency of intake, level of training, muscle group, or type of contraction (135). It may improve grip strength in older adults by accelerating muscle oxygenation and muscle strength recovery after exercise (136). Most of the current research suggests that a nitrate-rich diet has potential benefits for muscle strength and physical function in older adults, but due to the lack of research, more evidence is needed to validate this claim.

### 2.6. Probiotics, prebiotics, synbiotics

Probiotics are beneficial bacteria that are mainly found in our digestive system. Prebiotics are mainly oligosaccharides that promote the growth and proliferation of beneficial bacteria in the body but are not digested and absorbed by the host (137). Preparations that mix probiotics and prebiotics are called synbiotics (138), and the benefits of both are unified.

#### 2.6.1. Mechanisms

Changes in the structure of the intestinal flora are closely related to human health and disease. The major phyla of the healthy intestinal microbiota are the thick-walled phylum, the phylum Bacteroidetes, the phylum Actinomycetes, and to a lesser extent, the phylum Wolbachia and the phylum Aspergillus. In weak and cachectic humans, these beneficial bacteria are reduced, while an increase in opportunistic pathogens of Enterobacteriaceae occurs (139). Probiotics promote the production of metabolites such as short-chain fatty acids (SCFAs), secondary bile acids (BA), and some amino acids that regulate homeostasis in skeletal muscle by improving insulin sensitivity (140, 141). In addition, alterations in the ecosystem composition of the gut microbiota, such as reduced production of beneficial metabolites (e.g., SCFA) in the intestinal lumen, lead to intestinal leakage and bacterial endotoxins such as lipopolysaccharides (LPS) entering the peripheral blood (142), producing systemic inflammation associated with aging and muscle wasting (143, 144). Probiotics can limit inflammation and oxygen stress (145).

#### 2.6.2. Related studies

The experimental model of germ-free mice provides valuable evidence for the potential role of the microbiota in controlling muscle mass and function. Compared to conventional mice, germ-free mice, and antibiotic-treated mice, their muscle mass and strength are decreased (146–148). Interestingly, this change in muscle mass and function can be restored by transplantation of microbiota or under natural conditions (146, 147). The elderly were divided into high-functioning (HF) and low-functioning (LF) groups based on physical function, stool samples from both groups were transferred to germ-free mice, and grip strength was significantly increased in mice with HF compared to mice with LF (149). In mouse and human models, reduced intestinal permeability usually coincides with improved muscle mass or strength (150). The use of probiotics, prebiotics, and synbiotics may thus reduce muscle mass loss by stimulating the growth of the bacterial flora and restoring the balance of the gut microbiome, ultimately resulting in a more beneficial metabolite profile and lower intestinal permeability. COPD patients with sarcopenia who were continuously supplemented with a multistrain probiotic for 16 weeks showed reduced markers of intestinal permeability and neuromuscular junction degeneration in plasma, along with improved grip strength, gait speed, and SPPB scores compared to the placebo group (151). However, the causal relationship between microbiota and muscle health remains uncertain due to the lack of targeted studies and the effects of a large number of covariates (including diet, exercise, polydipsia, and multiple drugs) on microbiota composition and function (152). In addition, specific strains that optimize muscle mass and function are not yet available due to the scarcity of human studies and the difficulty of accurate measurements. Future studies should be conducted in humans and should focus on the effects of different bacterial genera and strains on microbiome balance, metabolite profiles, gut function, and muscle mass in sarcopenia.

### 2.7. Magnesium, selenium, calcium, and other minerals

Growing evidence shows that low micronutrient intake is associated with an increased risk of sarcopenia (153). It has been shown from systematic evaluations that patients with sarcopenia have lower intakes of calcium, magnesium, sodium and selenium than older adults with healthy muscles (154), and magnesium, selenium and calcium appear to be the most promising minerals for the prevention or treatment of sarcopenia (155).

Magnesium is involved in numerous physiological processes as a cofactor in many enzymatic reactions, and it also plays an important role in maintaining muscle mass and protecting muscle tissue from oxidative damage (156, 157). Mg^2+^ supplementation in aged mice induces myogenic differentiation, promotes protein synthesis, provides protection against the loss of muscle regeneration potential and muscle mass during aging, significantly promotes muscle regeneration, and preserves muscle mass and strength (158). The study suggests that intramuscular ionized magnesium is negatively correlated with age and positively correlated with the strength of knee extension in females. This may be because females have chronic underlying magnesium deficiency and therefore have significantly lower intramuscular ionized magnesium than males (159). In a cross-sectional study involving 2,570 women aged 18–79 years (156), a positive association between dietary magnesium intake and skeletal muscle mass and explosive leg strength index was observed, and data from another prospective cohort study suggested that higher magnesium intake is associated with greater grip strength and higher skeletal muscle mass (157). In follow-up surveys over 5 years, increased magnesium intake was associated with increased SPPB scores in older women, but no such association was observed in men (160). Higher intake of magnesium has been shown to be positively correlated with appendicular muscle mass and change in appendicular muscle mass in a longitudinal study (161), and positive associations between magnesium intake and grip strength have been shown in a cross-sectional study (162). There is some consistency in the current studies of magnesium's ability to improve sarcopenia, suggesting that magnesium supplementation may slow age-related skeletal muscle mass loss, although the evidence is mainly observational and cross-sectional studies.

Selenium is one of the essential trace elements, and it has been reported that patients with selenium deficiency develop skeletal muscle disease, manifested by muscle pain, fatigue, proximal limb weakness, and elevated serum creatine kinase (163). Although dietary supplements of selenium alone or in combination with vitamins are being widely used, the effects of selenium on muscle performance have not been adequately studied. Experiments conducted in mice show that selenium supplementation increases calcium release from the sarcoplasmic reticulum, thereby improving skeletal muscle performance, and that increased expression of selenoprotein N in muscle enhances oxidative stress tolerance (164). Selenium concentrations were found to be negatively associated with restricted physical function in a cross-sectional study, with a reduced incidence of physical frailty when baseline selenium levels were doubled (165). In the only clinical randomized controlled study, combined vitamin E, vitamin C, zinc, and selenium supplementation for 17 weeks improved maximal voluntary contraction and endurance limit time in the quadriceps muscle by reducing oxidative stress and enhancing antioxidant defense (166). Although selenium intake is low in elderly individuals and correlated with poorer skeletal muscle function, prospective analysis indicates no significant effect of selenium intake on skeletal muscle function (167). Nevertheless, the daily dietary intake of selenium is 20–75 μg for adults according to the EU recommendations (45). Since most of the evidence is from observational studies, we are not yet able to conclusively determine the effect of selenium supplementation in patients with sarcopenia; thus, large randomized controlled trials are required in the future to demonstrate this.

In the cross-sectional analysis, daily calcium intake was negatively correlated with overall fat percentage and positively correlated with extremity bone mass. After adjusting for age, sex, BMI, total energy intake, and lifestyle factors, daily calcium intake was significantly lower in patients with sarcopenia than in those without sarcopenia (168). However, 6 months of calcium supplementation does not have a significant effect on skeletal muscle strength and serum testosterone in young adult men, as found in one randomized controlled trial (169). There is a lack of studies on the effect of calcium on patients with sarcopenia.

### 2.8. Collagen and collagen peptides

Collagen accounts for one-third of the total protein in the human body, is the most abundant form of structural protein in the body, and contributes about 65%−80% of tendon dry weight (170). Extramyocellular connective tissue transmits contractility to tendons and bone, and collagen is a core structural component of extracellular connective tissue and is therefore essential for the strength, regulation, and regeneration of this tissue (171). Dietary collagens, such as collagen peptides or gelatin, are most commonly extracted from the skin, bones, or scales of pigs, cattle, and other poultry (172), and because they contain large amounts of glycine and proline and hydroxyproline, similar to the amino acid distribution of muscle connective tissue, it has been proposed that increasing their intake may help to stimulate muscle connective tissue synthesis to the greatest extent (173), thereby increasing muscle mass and strength and improving sarcopenia possibly.

#### 2.8.1. Mechanisms

Consumption of proline-rich and glycine-rich collagen may be more suitable than high-quality protein sources such as casein or whey protein (providing only 6% proline and 2% glycine) to provide specific amino acid precursors required to support *de novo* synthesis of connective tissue proteins since the amount of glycine and proline provided in the usual diet is insufficient to provide metabolism and promote increased rates of tissue collagen synthesis (174, 175). In an *in vitro* model, tendons in growth mediums containing proline and ascorbic acid showed increased collagen content and improved mechanical properties (176). In rats, a glycine-rich diet made the Achilles tendinitis model more resistant to maximum tolerated loads (177). In addition, peptides produced by collagen hydrolysis, which are easily absorbed in the digestive tract before entering the circulation (178), can enhance fibroblast elastin synthesis, while inhibiting elastin degradation and promoting fibroblast proliferation (179), and thus may enhance connective tissue remodeling in muscle.

#### 2.8.2. Clinical studies

Regarding the effect of collagen supplementation on body composition, Zdzieblik et al. (180) showed that elderly men with sarcopenia exercised three times a week and ingested 15 g of collagen peptide per day for 12 weeks, and their changes in body composition were very significant, with a mean increase in fat-free mass of 4.2 kg compared to only 2.9 kg in the placebo group. The same test in young, healthy men resulted in a mean increase in fat-free mass of 2.6 kg in the collagen peptide group and only 0.7 kg in the placebo-supplemented group (181). In premenopausal women, it was also found that the combination of resistance training with collagen supplementation significantly increased fat-free mass and increased hand grip strength. The above studies showed that collagen peptide supplementation was effective in improving muscle mass and strength while resisting resistance exercise. Regarding the effect of collagen on muscle protein synthesis, Oikawa et al. (182) supplemented 30 g of whey protein or collagen peptide twice a day in older adults who lacked physical activity and low-energy status, and only the whey protein group enhanced fat-free mass and muscle protein synthesis in the lower extremities during return to activity. Two other studies have similarly observed increased muscle protein synthesis with whey protein compared to collagen supplementation (183, 184). This suggests that collagen has little anabolic potential compared to isonitrogenous higher-quality protein sources. According to systematic reviews and meta-analyses, collagen supplements are most beneficial in reducing joint pain and improving joint function, with some improvement in body composition, strength, and muscle recovery (170). In conclusion, collagen supplementation with resistance exercise can increase muscle mass and strength, but there is insufficient evidence that collagen is more effective in improving sarcopenia than traditional high-quality protein sources such as casein or whey protein.

### 2.9. Polyphenols

Polyphenols are a range of plant compounds with antioxidant and anti-inflammatory properties containing one or more phenolic rings attached to hydroxyl groups (185). They are divided into four classes: phenolic acids, flavonoids, stilbenes, and lignans (186) and are particularly abundant in fruits, vegetables, coffee, tea, cocoa, vanilla, and spices (187). Because there are a wide variety of polyphenols available and there are many factors that can alter their concentration in food, it is difficult to establish reference composition tables (188).

#### 2.9.1. Mechanisms

The effects of polyphenolic compounds in dystrophia are mainly through the inhibition of E3 ubiquitin ligases and upstream regulators in inflammation, oxidative stress, and mitochondrial damage (189, 190). It also increases protein synthesis by effectively activating the Akt/mTOR pathway (191). Moreover, PPs modulated the expression of miRNAs, IGF-1 signaling pathway, follistatin, mitochondrial biogenesis, and myogenic differentiation factors involved in myogenesis (192).

#### 2.9.2. Related studies

Resveratrol (RSV) is a natural polyphenol. In animal experiments, high doses of RSV (400 mg/kg/day) have been reported to attenuate muscle fiber atrophy following hindlimb suspension in rodents (193). Lower doses of RSV (5 mg/kg/day) still promoted skeletal muscle hypertrophy and reduced exercise-induced muscle necrosis in wild-type mice (194). In clinical studies, elderly subjects were supplemented with 500 mg/day resveratrol during exercise, and muscle mitochondrial density and muscle fatigue resistance were higher in elderly subjects compared with placebo-supplemented groups (195). Resveratrol at 1,000 mg/day increased the 6-min walk distance by 33.1 m in older adults, which was higher than the mean walking distance in the 500 mg/day group (196). Patients with chronic kidney disease received 500 mg resveratrol and 500 mg curcumin orally daily, and muscle mass and bone mass increased significantly after 12 weeks. However, no improvement in walking ability with resveratrol was observed in elderly subjects with peripheral arterial disease (197), mitochondrial function in skeletal muscle was not improved and lean body mass was decreased in COPD patients receiving 150 mg/day resveratrol (198). To determine the effects of polyphenols on muscle, multiple systematic reviews and meta-analyses have assessed the effectiveness of polyphenols on muscle pain and muscle recovery after exercise in healthy adults, and the results have shown that consumption of polyphenol-rich foods, juices, and concentrates accelerates the recovery of muscle function and reduces muscle soreness at doses ranging from 150 to 1,500 mg/day (199–201). A meta-analysis suggests that polyphenol supplementation is unlikely to enhance exercise-induced changes in body composition or performance, and that only isoflavones may increase lean body mass in postmenopausal women (202), and another meta-analysis suggests that short-term polyphenols intake, although attenuating the inflammatory response after exercise, does not affect the anabolic response to protein and exercise in healthy elderly men (203). In summary, polyphenol supplementation is believed to reduce muscle pain and accelerate the recovery of muscle function after exercise, but the effect on body composition and physical performance in patients with sarcopenia is inconclusive and remains to be explored.

## 3. Conclusions

Clinicians or health care providers need to screen older adults at risk for sarcopenia, especially those with comorbid malnutrition, and use appropriate diagnostic tools to make the diagnosis. Related professionals should then provide resistance training and diet and protein optimization programs to patients with diagnosed sarcopenia (15). This article summarizes the research progress of nutritional supplements in the improvement of sarcopenia, including the possible cellular and molecular mechanisms involved, so as to provide a reference for medical staff and researchers. The currently acceptable recommended intakes for each nutrient are shown in Table 2.

**Table 2:** Nutrients that may improve sarcopenia and recommended intake.

**Nutrients**	**Recommended intake dose**
Protein	1–1.2 g/kg/day for healthy elderly, 1.2–1.5 g/kg/day for the malnourished, or 25–30 g protein per meal (36)
Leucine	2.5–2.8 g per meal (36)
HMB	3 g/day (40)
Vitamin D	800–1,000 IU/day (41)
Vitamin E	400 IU/day (42)
Vitamin C	45 to 90 mg/day (43)
Magnesium	300 mg/day for men and 270 mg per day for women (44)
Selenium	25–75 μg/day (45)
Calcium	1,000–1,200 mg/day (46)
Probiotics	400 μg/day (47)
Inorganic nitrate	3.7 mg/kg/day (48)
Collagen	50 mL/day (49)
Polyphenols	>500 mg (50)

In the future, patients may benefit from complex hybrid nutritional supplements, as well as the development of nutrigenomics and metabolomics (204), so that nutritional interventions provided are tailored to an individual's nutritional and metabolic status. In addition, when the molecular mechanisms of muscle targets are well studied, they may play a key role in developing targeted treatment and prevention strategies.

## Author contributions

SLiu contributed to drafting the paper. LZ had primary responsibility for final content. LZ and SLi revised the final draft of the manuscript. All authors read and approved the final manuscript.
